# HEALTHCARE UTILIZATION IN PATIENTS WITH SPINA BIFIDA AND BONE FRACTURES

**DOI:** 10.2340/jrm.v58.45674

**Published:** 2026-06-30

**Authors:** Adrian M. FERNANDEZ, Than KYAW, Debbie GOLDBERG, I. Elaine ALLEN, Yi LI, Alfredo FERNANDEZ, Hillary L. COPP, Lindsay A. HAMPSON

**Affiliations:** 1 Department of Urology, University of California, San Francisco, San Francisco, CA; 2 Massachusetts General Hospital, Boston, MA; 3 Department of Epidemiology and Biostatistics, University of California, San Francisco, San Francisco, CA; 4 Peninsula Orthopedic Associates, Daly City, CA, USA

**Keywords:** bone fracture, healthcare utilization, spina bifida

## Abstract

**Objective:**

To compare bone fractures and fracture-related healthcare costs in individuals with and without spina bifida (SB).

**Design:**

This is a retrospective observational trial.

**Subjects/Patients:**

California’s Healthcare Access and Information database was utilized to identify all individuals with SB who sought care in California emergency departments, inpatient hospitals, or ambulatory surgery centres between 2005 and 2017.

**Methods:**

Bone fracture encounters and associated healthcare utilization factors were compared among individuals with spina bifida vs individuals without spina bifida, matched 5:1 by birth year.

**Results:**

Between 2005 and 2017, 20,290 individuals with spina bifida sought care in California emergency departments, inpatient hospitals, or ambulatory surgery centres. Compared with 101,450 individuals in the comparison group, those with spina bifida were more likely to seek care for a bone fracture (OR 1.64, *p* < 0.001) and more likely to sustain repeat fractures (36% vs 26%, *p* < 0.001). Individuals with spina bifida sustained more bone fractures per capita at each body location studied, with more than double the fractures per capita at the chest (2.0x), foot (2.1x), pelvis (2.4x), leg (2.5x), and vertebrae (2.5x). Individuals with spina bifida spent more cumulative days hospitalized for bone fractures (mean 9.4 vs 7.6, *p* < 0.001).

**Conclusion:**

Individuals with spina bifida were diagnosed with more bone fractures per capita than age-matched persons without spina bifida.

## LAY ABSTRACT

Using California’s Healthcare Access and Information database, we identified all individuals with spina bifida who sought care in California emergency departments, inpatient hospitals, or ambulatory surgery centres between 2005 and 2017. We also generated a comparison group of individuals without spina bifida, matched by birth year. Among the 2 groups, we compared bone fractures and other healthcare utilization factors related to bone fractures, such as cost of care and duration of hospitalization. We found that individuals with spina bifida were more likely to seek care for bone fracture, experience repeat fractures, and spend time in the hospital as a result of broken bones. People with spina bifida had more bone fractures at each body area evaluated, especially the chest, foot, pelvis, leg, and vertebrae. While other studies have reported high incidences of fractures in people with spina bifida, this population-level comparison is novel, demonstrating the importance of future work to prevent fractures in this vulnerable population.

Spina bifida (SB), conventionally considered a medical condition portending a relatively shorter lifespan for the affected individual, has become a manageable chronic disease now associated with a lifespan duration similar to that enjoyed by unaffected persons. However, as people with SB live longer, the healthcare challenges facing these individuals into adulthood remain poorly understood. The failure of closure of the caudal spinal cord *in ute*ro can have many compounding downstream effects, contributing to high healthcare utilization for the duration of an individual’s life (1, 2).

Those with SB bear risk to the skeletal system. Bone mass, typically accumulated as a result of local stresses, strains, and mechanical loads, may be reduced in individuals with SB, particularly in those with limited ambulatory status (3–5). In many cases, those with SB have severe motor and sensory problems. Chronic kidney disease and hydrocephalus are broadly prevalent among persons with spina bifida; those and similar diseases may have contributed to inhibited bone growth and mineralization (6–9). However, population-based studies comparing bone fractures among individuals with SB and those without are sparse.

The current study captures bone fracture encounters at California-licensed emergency departments (EDs), hospitals, and ambulatory surgery centres (ASCs) from 2005 to 2017 to examine differences in fracture types, treatment modalities, and healthcare utilization among California residents with SB vs a birth-year-matched comparison group. We hypothesized there would be no difference between the 2 groups in fracture incidence and hospitalization costs. If that null hypothesis were to be rejected, we might have evidence that bone fractures and treatment encounters occur more frequently among persons with SB than among age-matched members of a comparison group.

## METHODS

### Data source

The California Department of Healthcare Access and Information, previously the Office of Statewide Health Planning and Development, collects patient-level administrative data from all hospital inpatient, outpatient surgery, and ED encounters in the state. An “encounter” refers to a billable event at an ED or ASC or a discharge from an inpatient hospital. The database includes prima facie abstracted data from individual patient records (birthdate, diagnoses, treatments/procedures performed, charges, and discharge disposition), which are available for research and public health purposes upon request (10). In circumstances in which an individual is admitted to the hospital from an ED, the encounter is documented only as an inpatient encounter to avoid duplication.

### Participant selection

Individuals with SB were identified using relevant primary and non-primary diagnoses listed in their electronic medical record, including SB diagnosis codes (ICD-9-Clinical Modification or ICD-10-Clinical Modification) or myelomeningocele repair codes (ICD-9-Clinical Modification or CPT) at healthcare encounters among any California ED, inpatient hospital, or ASC from January 1, 2005 to December 31, 2017. The list of ICD and CPT codes utilized for SB diagnosis is available in Table SI.

A cohort of persons without SB (the comparison group) was created by random selection of individuals without SB who were treated at any California ED, inpatient hospital, or ASC between 2005 and 2017. Comparison group individuals were frequency matched 5:1 by year of birth with individuals from the SB group.

All individuals were tracked during the study period using record linkage numbers (RLN). Those with missing RLN were excluded to prevent case duplication. Individuals with missing birthdate or non-California addresses were also excluded.

### Primary outcomes: fracture encounters, episodes, and treatment

Fracture diagnosis or procedure codes were used to identify patients with bone fractures (see Table SI). In the absence of a fracture diagnosis code, patients were considered to have a fracture if they underwent a fracture-specific treatment (e.g., CPT-4 25652 open treatment of *ulnar styloid fracture*).

Fracture *encounters* were any ED, inpatient hospital, or ASC visit in which a fracture diagnosis or procedure code was documented. A fracture *episode* was defined to identify “new” fractures sustained by a particular individual (i.e., a discrete fracture). A fracture episode was therefore defined as any fracture occurring in a body part that had not previously been fractured, or a fracture diagnosis in a body part previously fractured only if 6+ months (180+ days) had elapsed since the original fracture diagnosis encounter. Any fracture diagnosis in the same body part within 6 months was not considered a new “episode”.

### Predictor variables

*Demographic variables.* The demographic variables evaluated in this study include age of the patient at the time of their encounter, biological sex, race, and ethnicity, all as defined in HCAI.

*Socioeconomic variables.* Socioeconomic status (SES) was analysed by creating neighbourhood scores at the zip code tabulation area (ZCTA) of residence using US census data (11). Each patient was assigned a corresponding neighbourhood score derived from the Diez-Roux method based on their ZCTA of residence. Quartiles were then created based on the distribution of neighbourhood scores among the comparison group of patients, with the 1^st^ quartile of the Diez-Roux representing the lowest SES and the 4th quartile representing the highest SES (12, 13).

*Healthcare-related variables.* Insurance status, classified as private, non-private, mixed, and unknown, were identified. Health professional shortage areas (HSPA), which is defined as areas having a shortage of medical providers, were also incorporated into our analysis as quartiles based on the distribution among the comparison group (i.e., the lowest 25% of lowest shortage and the highest 25% of highest shortage). Hospital length of stay (days) and charge ($) were reported for each patient’s in-hospital health encounters. Disposition was reported for each encounter and after-care was categorized into home, home health service, skilled nursing or intermediate care, death, and other.

*Health related variables.* The Charlson Comorbidity Index (CCI), which is a weighted index to predict death within a year of hospitalization, was used to objectively assess each individual’s risk of mortality in relation to a list of specific comorbidities. For this study, any 1 of 17 comorbid conditions (including congestive heart failure, diabetes mellitus, dementia, and others) were tagged and assigned a weight from 1 to 6 for each individual based on the estimated 1-year mortality hazard ratio from a Cox proportional hazards model. The sum of the weighted values comprised the individual’s final CCI score (14, 15).

### Statistical analyses

Given the large sample size, *p* < 0.01 was considered statistically significant. Descriptive analyses comparing characteristics between individuals with SB and non-SB persons utilized the Pearson’s χ^2^ test for comparing frequencies and the 2-tailed independent groups Student’s *t*-test for comparing means. Continuous variables were reported as means and standard deviations whereas categorical variables were reported as frequencies and percentages.

Univariate and multivariate logistic regression analyses were employed to adjust for predictor variables and describe the extent of utilization between the 2 groups. The outcome was the presence of 1+ bone fracture episode – allowing for assessment of the odds ratio (OR) and 95% confidence interval (CI) for a bone fracture. Predictor variables were analysed in separate univariate models and were included in the final multivariable model if *p* < 0.01. All analyses were performed with SAS, version 9.4 (SAS Institute, Cary, NC, USA).

## RESULTS

Between 2005 and 2017, an estimated 20,290 individuals with SB sought care in California EDs, inpatient hospitals, or ASCs. Compared with a birth-year-matched comparison population, those with SB were more likely to seek care for a bone fracture (18% SB vs 11% comparison group, *p* < 0.001) (Table I).

**Table I:** Sociodemographic characteristics of individuals with SB vs comparison group in California, 2005–2017

Characteristics	SB		Comparison group		*p*-value*
	*n*	%	*n*	%	
Total # of individuals	20,290	100	101,450	100	
# individuals with ≥1 fracture	3,551	18	11,627	11	< 0.001
Sex					< 0.001
Male	8,239	40	47,757	47	
Female	11,490	57	51,864	51	
Unknown	561	3	1,829	2	
Race					< 0.001
White/Caucasian	9,156	45	51,312	51	
Black/African American	952	5	7,405	7	
Asian/Pacific Islander	571	3	8,286	8	
Native American/Other	1,663	8	13,528	13	
Multiracial	7,907	39	19,298	19	
Unknown	41	< 1	1,621	2	
Ethnicity					< 0.001
Hispanic	4,957	25	27,733	27	
Non-Hispanic	797	53	61,053	60	
Mixed	4,327	21	10,718	11	
Unknown	209	1	1,946	2	
Insurance					< 0.001
Non-private	8,723	43	38,880	38	
Private	3,521	17	37,074	37	
Mixed	8,032	40	25,238	25	
Unknown	14	< 1	258	< 1	
Charlson Comorbidity Index					< 0.001
0	8,601	42	74,013	73	
1	5,698	28	16,864	16	
2	2,604	13	4,692	5	
3+	3,387	17	5,881	6	
Mean Charlson Comorbidity Index (weighted) (SD)	1.8 (2.6)		0.7 (1.8)		< 0.001
Neighbourhood measures					
Health Professional Shortage Area Score					< 0.001
Q1 (low shortage)	6,991	35	21,414	21	
Q2	4,551	22	24,817	24	
Q3	4,096	20	27,208	27	
Q4 (high shortage)	4,334	21	24,867	25	
Unknown	318	2	3,144	3	
Neighbourhood SES (Diez-Roux)					< 0.001
Q1 (low SES)	4,837	24	24,191	24	
Q2	4,830	24	22,308	22	
Q3	5,337	26	22,652	22	
Q4 (high SES)	4,560	22	26,150	26	
Unknown	726	4	6,149	6	

* Please note that, on account of the large sample sizes, certain comparisons (such as the neighbourhood SES distributions) may yield a statistically significant difference without clear substantive difference.

Compared with persons with SB, those without SB were more likely to be male (47 vs 40%, *p* < 0.001), of white race (51 vs 45%, *p* < 0.001), and privately insured (37 vs 17%, *p* < 0.001). Mean CCI scores were higher for individuals with SB (1.8) vs the comparison group (0.7, *p* < 0.001). A higher proportion of individuals with SB lived in areas of low physician shortage (35 vs 21%, *p* < 0.001), and SES measures, though statistically significantly different, were overall quite similar between groups based on Diez-Roux scoring (see Table I).

Comparing those in each group who sustained 1 or more fractures, persons with SB had higher mean fracture encounters (1.8 vs 1.4, *p* < 0.001) over the study period (Table II). Those with SB were more likely to sustain 2 or more fracture episodes within the study period (36% SB vs 26% non-SB, *p* < 0.001). Those with SB had more surgical encounters overall (1.2 SB vs 1.1 comparison group, *p* = 0.005 (Table II).

**Table II:** Bone fractures and fracture-related healthcare utilization among individuals with SB vs persons without SB

Characteristics	SB with 1+ fracture episode		Comparison group with 1+ fracture episode		*p*-value
	*n*	%/SD	*n*	%/SD	
Total # of individuals	3,551		11,627		
Total # of health encounters	112,867		109,842		< 0.001
Fracture encounters					
Total # (%)	6,256	6%	16,472	15%	
Mean (SD)	1.8	2.2	1.4	0.9	< 0.001
Fracture episodes					
Total # (%)	5,833	5%	16,417	15%	
Mean (SD)	1.6	1.2	1.4	0.9	< 0.001
Individuals with 2+ fracture episodes	1,276	36%	3,059	26%	< 0.001
Setting of fracture episode					
ED	2,111	36%	4,308	26%	< 0.001
Inpatient	3,518	60%	11,343	69%	< 0.001
Ambulatory Surgical Centres	204	4%	766	5%	< 0.001
Fracture-treatment Encounters					
Total # (%)	1,008		3,152		0.005
Mean (SD)	1.2	0.5	1.1	0.4	0.005
Fracture-related length of hospitalization (LOS) & costs					
Mean (SD)	6.5	13.4	5.8	10.5	0.063
Cumulative LOS (SD)	9.4	20.8	7.6	14.5	0.002
Mean US$ charge per hospitalization (SD)	97K	165K	92K	182K	0.36
Mean US$ charge for cumulative hospitalization (SD)	133K	240K	115K	225K	0.019

**Fig. 1:**
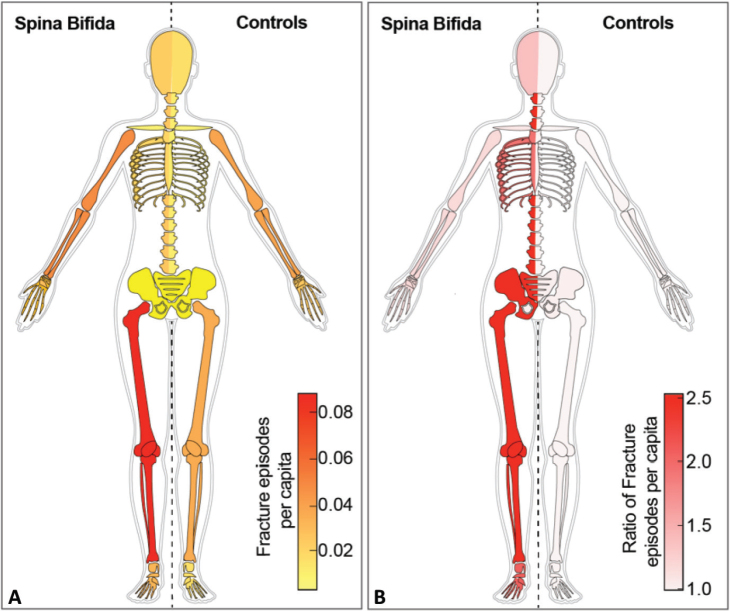
(A) Heat maps of fracture episodes per capita and (B) ratio of fracture episodes per capita showing differences between individuals with spina bifida and a comparison group of persons without SB.

Individuals with SB sustained more bone fractures per capita at each body location studied (Table III), with more than double the fractures per capita at the chest (2.0x), foot (2.1x), pelvis (2.4x) leg (2.5x), and vertebrae (2.5x) (Fig. 1).

**Table III:** Bone fracture episodes across specific body locations comparing spina bifida and individuals without SB

Individuals with ≥ 1 specific fracture diagnosis						
Fracture body locations	SB			Comparison group		
	Patients, *n*	Fracture episodes, *n*	Fracture episodes, %	Patients, *n*	Fracture episodes, *n*	Fracture episodes, %
Arm	865	953	16.3%	3,660	3,894	23.7%
Chest	342	377	6.5%	899	937	5.7%
Clavicle	132	137	2.3%	599	617	3.8%
Foot	597	636	10.9%	1,452	1,490	9.1%
Hand	525	569	9.8%	2,286	2,414	14.7%
Head	394	437	7.5%	1,452	1,518	9.2%
Leg	1,513	1,779	30.5%	3,305	3,524	21.5%
Other	283	309	5.3%	729	753	4.6%
Pelvis	118	126	2.2%	259	266	1.6%
Vertebrae	450	510	8.7%	916	1,004	6.1%

Individuals with SB had higher odds of sustaining a bone fracture in univariate analysis vs the comparison group (OR 1.64, *p* < 0.001). This remained true in multivariable analysis, controlling for comorbidities and socioeconomic factors (OR 1.19, *p* < 0.001) (Table SII).

For each inpatient hospital encounter related to a fracture, individuals with SB averaged more days in hospital vs the comparison group (6.5 vs 5.8 days, *p* = 0.063). Charges for an individual fracture hospitalization were minimally higher ($97K) in the SB group vs the comparison group ($92K, *p* = 0.360). Among those with fractures, the average number of cumulative inpatient days for bone fractures was 9.4 days among SB individuals vs 7.6 among the comparison group (0.002). Cumulative charges were overall higher for SB fracture hospitalizations, though not statistically significant (133K vs 115K, *p* = 0.019) (see Table II).

In terms of disposition, a slightly smaller proportion of individuals with SB were discharged home after inpatient hospitalization with a fracture diagnosis vs the comparison group (92% SB vs 95% comparison, *p* < 0.001). More SB patients required home health services (2% SB vs 1% comparison group) and slightly more were discharged to a skilled nursing facility (SNF) or intermediate care home compared with controls (4% vs 2%, *p* < 0.001) (Table IV).

**Table IV:** Discharge disposition after fracture encounter comparing spina bifida and comparison group by encounter

Disposition	SB		Comparison group		*p*-value
	*n*	%	*n*	%	
Routine home	18,696	92	96,632	95	< 0.001
Home health service	454	2	769	1	< 0.001
Skilled nursing, intermediate care	711	4	1,889	2	< 0.001
Died	35	< 1	202	< 1	< 0.001
Other	394	2	1,958	2	< 0.001

## DISCUSSION

For decades, case series from single institutions across the globe have identified a high rate of bone fractures among individuals with SB (16–20). However, population-level data comparing bone fractures and associated healthcare utilization factors in SB vs the general population have been lacking. We studied California’s HCAI database to understand how individuals of all ages with SB have utilized healthcare for bone fractures in comparison with persons without SB, finding that individuals with SB experienced more bone fractures per capita when compared with an age-matched comparison group. Furthermore, these increases in fractures were within all body parts investigated, especially at the lower extremities, chest, vertebrae, and pelvis. Bone fractures in individuals with SB were more likely to be diagnosed in an inpatient hospital setting and less likely to have fractures diagnosed in the ED compared with those without SB. Length of stay and hospitalization-related charges were higher for bone fracture treatment among those with SB, cumulatively.

The increased overall burden of bone fractures among those with SB may be related to systemic problems, such as endocrine and electrolyte changes, affecting people with SB. Hydrocephalus, for example, present in approximately 80–90% of individuals with SB, can result in changes of the hypothalamic-pituitary axis, leading to deficiencies in growth hormone secretion and stunted bone growth (7, 9). Early development of chronic kidney disease, common in those with SB due to complications of neurogenic bladder, can lead to renal osteodystrophy and chronic metabolic acidosis, ultimately demineralizing bone (21, 22). The combined effect of these systemic factors might explain the increased numbers of bone fractures per capita in SB across *all* body parts studied, including body parts that are not neurologically impacted by the myelomeningocele.

The lower extremity sensory and motor compromise from SB may explain the especially high numbers of bone fractures seen in the lower limbs of those with SB. Smaller case series have noted a high incidence of lower extremity fractures in SB (18, 19, 23), and our data demonstrate more than double the fractures per capita among those with SB vs the comparison group at the leg (2.5x), pelvis (2.4x), and foot (2.1x) (see Fig. 1). Bone mass is typically accumulated as a result of cumulative stresses, strains, and mechanical loads on the skeleton (3–5, 7). The lower ambulatory ability of many individuals with SB can compromise bone architecture and strength, predisposing the lower limbs to low-trauma fractures. This is a similar pattern of bone fractures to that identified in individuals with spinal cord injury, who are at risk of sub-lesional osteoporosis, like persons with SB (24).

The sensory deficits to the lower extremities in SB may also explain the differences in hospital settings where fracture diagnoses occur. A higher percentage of fractures were identified in the inpatient setting in SB vs the general population, whereas a higher proportion of fractures were diagnosed in the ED among the comparison group. Individuals with SB are liable to sustain “silent” fractures owing to sensory deficits, and bone injuries can therefore go undiagnosed (5, 7, 18). Perhaps the high incidence of bone fractures diagnosed in inpatients reflects a higher number of incidental bone fractures in those with SB compared with the more symptomatic fractures in the general population.

Fractures among individuals with SB might also be attributed to socioeconomic or environmental factors. Certain data suggests that low education levels and low income are associated with lower levels of bone mineral density in the general population, though this has not been studied to our knowledge in those with SB (25). Our study did not demonstrate a meaningful difference between SES characteristics among those with SB vs the comparison group (see Table I). This is not completely consistent with prior studies, however, some of which have identified SES differences among those with SB, such as maternal education level (26). As the SES characteristics of each group were very similar in this analysis, the current study does not support SES differences as a major contributor to the higher number of fractures seen in the SB group. Additional research should explore the non-medical contributors to bone fractures among individuals with SB.

Our findings demonstrate that bone fractures contribute to higher healthcare utilization among individuals with SB. While the cumulative length of hospitalization for bone fracture treatment was significantly longer for those with SB (9.4 vs 7.6 days), the differences in healthcare costs related to fracture treatment are modest ($133K vs $115K). The differences in discharge disposition are also small (92% discharge to home in the SB group vs 95%). Though these differences are statistically significant, they may not be clinically meaningful or may reflect the overall higher comorbidity state of the individuals with SB as demonstrated by the higher average CCI scores in this group.

Regardless, efforts aimed at promoting bone mineral density and reducing fractures may reduce morbidity, healthcare utilization, and healthcare costs among individuals with SB. More work is necessary to study bone strengthening for people with SB, but treatments such as bisphosphonate therapy may be a promising first option for fracture prevention (27, 28). Periods of cast immobilization have previously been demonstrated as a high risk time for repeat bone fractures in SB (18, 20, 29), so perhaps bisphosphonate therapy should be studied to prevent repeat fractures at times when cast immobilization is necessary.

### Limitations

Limitations to this study include those anticipated in secondary database analysis, namely the lack of detailed clinical information provided regarding each hospital encounter. Procedural and diagnostic codes do not necessarily indicate a true diagnosis, even with highly specific coding. Additionally, demographic designations such as race and ethnicity may be inaccurate or incomplete, especially in large datasets such as HCAI, and therefore adjustment around these potential confounders should be interpreted cautiously. Instead of reporting specific bones fractured (e.g., “fibula” or “ulna”), we grouped body areas (e.g., “leg” and “arm”) together because codes were often non-specific (e.g., M84.639S “pathological fracture of unspecified ulna and radius, subsequent”). Additionally, as diagnoses were included only in California, we may have missed fracture episodes for those that moved into or out of the state. Some individuals likely moved into and out of California over the course of the study period, and therefore the amount of observation time within the HCAI database was not necessarily equal across groups, despite the frequency matching on birth year. It is also possible that individuals sustained fractures for which they did not seek care. This may be especially true in the SB population, as bone fractures in this group can be asymptomatic and therefore go undiagnosed (5, 7). The focus on encounters in the state of California may limit generalizability. The large group sizes in the current study allow for small differences between groups to be statistically significant, even though these differences may not be clinically meaningful. Finally, the 6-month (180 day) cutoff used to define fracture “episodes” was determined based on expert opinion instead of a particular biological/pathological process. This cutoff prevents us from diagnosing multiple discrete fractures in the same body area within 6 months and risks re-counting fractures with prolonged healing times. Though the ICD-10-CM “7th character” can differentiate “initial” vs “subsequent” encounters for certain fractures, this does not apply for the HCAI data recorded before October 2015, as fractures were coded using ICD-9-CM. In future work that does not include ICD-9-CM coding, perhaps the 7^th^ character of ICD-10-CM coding can be used as a more reliable marker to delineate discrete fractures.

### Conclusions

This study demonstrates that individuals with SB in California were diagnosed with more bone fractures per capita than persons in the age-matched comparison group. Bone fractures of the lower extremities were more prevalent among those with SB, possibly because of neuromuscular compromise to the lower body. Bone fractures contribute to high healthcare utilization among individuals with SB, and efforts to prevent fractures in this vulnerable population should be studied.

## Supplementary Material




