# Supplementary material for: HEALTHCARE UTILIZATION IN PATIENTS WITH SPINA BIFIDA AND BONE FRACTURES
Source: J Rehabil Med. 2026 Jun 30;58:45674. doi: 10.2340/jrm.v58.45674 (PMC13329719; doi:10.2340/jrm.v58.45674)
Supplement: Supplementary file 2 [file JRM-58-45674-s2.pdf]

#SPILL!

Characteristic	Categories	Outcome: Fracture encounters 1+ fracture encounters %	Univariate logistic regression OR (95% CI)	p-value	MV logistic regression OR (95% CI)	p-value
**Patient type**						
	SB	23	1.64 (1.57, 1.71)	**<0.001**	1.19 (1.14, 1.24)	**<0.001**
	Comparison	77	ref		ref	
**Sex**						
	Male	55	ref		ref	
	Female	42	0.64 (0.62, 0.66)	**<0.001**	0.60 (0.58, 0.63)	**0.004**
	Unknown	3	1.32 (1.19, 1.46)	**<0.001**	0.81 (0.73, 0.90)	**<0.001**
**Race**						
	White	50	ref		ref	
	Black	6	0.78 (0.72, 0.84)	**<0.001**	0.71 (0.66, 0.77)	**<0.001**
	Asian/PI	4	0.48 (0.44,0.52)	**<0.001**	0.53 (0.48, 0.58)	**<0.001**
	Other	8	0.58 (0.54, 0.62)	**<0.001**	0.74 (0.69, 0.79)	**<0.001**
	Multiracial	32	1.49 (1.43, 1.55)	**<0.001**	1.20 (1.15, 1.26)	**<0.001**
	Unknown	<1	0.35 (0.28, 0.44)	**<0.001**	0.59 (0.45, 0.76)	**<0.001**
**Ethnicity**						
	Hispanic	22	0.83 (0.79, 0.86)	**<0.001**	0.78 (0.74, 0.82)	**<0.001**
	non-Hispanic	58	ref		ref	
	Mixed	19	1.76 (1.69, 1.85)	**<0.001**	1.18 (1.12, 1.25)	**<0.001**
	Unknown	1	0.38 (0.31, 0.46)	**<0.001**	0.61 (0.49, 0.76)	**<0.001**
**Insurance**						
	non-Private	38	1.60 (0.53, 1.67)	**<0.001**	1.42 (1.35, 1.49)	**<0.001**
	Private	21	ref		ref	
	Mixed	40	2.58 (2.47, 2.70)	**<0.001**	1.94 (1.85, 2.04)	**<0.001**
	Unknown	<1	0.44 (0.23, 0.83)	**0.011**	0.51 (0.27, 0.96)	**0.036**
**Charlson comorbidity**	continuous		1.15 (1.14, 1.15)	**<0.001**	1.09 (1.08, 1.10)	**<0.001**
**Disposition at 1st encounter**						
	Routine home	93	ref		ref	
	Home health service	1	1.53 (1.32, 1.78)	**<0.001**	1.19 (1.02, 1.39)	**0.027**
	Skilled nursing/intermediate care/other	3	1.80 (1.63, 1.98)	**<0.001**	1.35 (1.22, 1.49)	**<0.001**
	Died	<1	0.77 (0.50, 1.19)	**0.241**	0.72 (0.46, 1.10)	0.13
	Other	2	1.24 (1.10, 1.39)	**<0.001**	1.05 (0.93, 1.18)	0.46
**Physician shortage (HPSA)**						
	Q1 (low shortage)	25	ref		ref	
	Q2	25	0.98 (0.93, 1.02)	0.31	1.01 (0.96, 1.06)	0.846
	Q3	25	0.87 (0.83, 0.91)	**<0.001**	0.92 (0.87, 0.97)	**0.001**
	Q4 (high shortage)	24	0.90 (0.86, 0.95)	**<0.001**	0.92 (0.88, 0.97)	**0.002**
	Unknown	<1	0.45 (0.39, 0.52)	**<0.001**	0.64 (0.54, 0.76)	**<0.001**
**DiezRoux SES**						
	**Q1 (low SES)**	24	1.08 (1.03, 1.14)	**0.002**	1.02 (0.96, 1.07)	0.565
	**Q2**	24	1.20 (1.15, 1.26)	**<0.001**	1.06 (1.01, 1.12)	0.025
	**Q3**	25	1.21 (1.15, 1.27)	**<0.001**	1.08 (1.02, 1.13)	**0.004**
	**Q4 (high SES)**	23	ref		ref	
	**Unknown**	4	0.73 (0.66, 0.80)	**<0.001**	0.90 (0.81, 1.01)	0.086
